# Systematic Evaluation of *Drosophila* CRISPR Tools Reveals Safe and Robust Alternatives to Autonomous Gene Drives in Basic Research

**DOI:** 10.1534/g3.115.019083

**Published:** 2015-05-20

**Authors:** Fillip Port, Nadine Muschalik, Simon L. Bullock

**Affiliations:** Cell Biology Division, MRC-Laboratory of Molecular Biology, Francis Crick Avenue, Cambridge CB2 0QH, United Kingdom

**Keywords:** *Drosophila*, CRISPR, mutagenesis, homology-directed repair, gene drive

## Abstract

The Clustered Regularly Interspaced Short Palindromic Repeat/CRISPR associated (CRISPR/Cas) technology allows rapid, site-specific genome modification in a wide variety of organisms . Proof-of-principle studies in *Drosophila melanogaster* have used various CRISPR/Cas tools and experimental designs, leading to significant uncertainty in the community about how to put this technology into practice. Moreover, it is unclear what proportion of genomic target sites can be modified with high efficiency. Here, we address these issues by systematically evaluating available CRISPR/Cas reagents and methods in *Drosophila*. Our findings allow evidence-based choices of Cas9 sources and strategies for generating knock-in alleles. We perform gene editing at a large number of target sites using a highly active Cas9 line and a collection of transgenic gRNA strains. The vast majority of target sites can be mutated with remarkable efficiency using these tools. We contrast our method to recently developed autonomous gene drive technology for somatic and germline genome engineering and conclude that optimized CRISPR with independent transgenes is as efficient, more versatile, and does not represent a biosafety risk.

The Clustered Regularly Interspaced Short Palindromic Repeat/CRISPR associated (CRISPR/Cas) genome engineering system is currently revolutionizing experimental and applied biology (Hsu *et al.* 2014; Doudna and Charpentier 2014). The system consists of the bacterial endonuclease Cas9 and a small chimeric guide RNA (gRNA), which directs Cas9 to a genomic target site 5′ to an NGG protospacer adjacent motif (PAM) (Jinek *et al.* 2012). Repair of CRISPR/Cas-mediated DNA double-strand breaks by endogenous proteins can result in insertions and deletions (indels) that disrupt gene function or—together with a supplied piece of donor DNA—precise sequence alterations. This methodology has been used successfully to edit the genome of a variety of organisms, although mutagenesis efficiencies have varied widely between different target sites, even in individual studies.

Several studies have demonstrated the feasibility of CRISPR/Cas genome engineering in the soma and the germline of *Drosophila melanogaster*, a major model organism in biomedical research (Bassett *et al.* 2013; Gratz *et al.* 2013, 2014; Sebo *et al.* 2013; Kondo and Ueda 2013; Ren *et al.* 2013, 2014; Yu *et al.* 2014; Port *et al.* 2014; Gokcezade *et al.* 2014; Zhang *et al.* 2014). These proof-of-principle studies have suggested a number of strategies to modify the fly genome, differing mostly in the way the Cas9 and gRNA are delivered. A popular method is centered on transgenic *cas9 Drosophila* strains, which are injected either with gRNA-encoding plasmids (Ren *et al.* 2013; Gratz *et al.* 2014) or crossed to strains with gRNA transgenes (Kondo and Ueda 2013; Port *et al.* 2014; Chen *et al.* 2015) [we now refer to this latter method as CRISPR with independent transgenes (CRISPR-it)]. These methods benefit from high success rates, easy generation of the necessary reagents, and the possibility to outsource gene targeting to commercial providers. A number of different transgenic *cas9* strains have been described and are publicly available. However, because there has been no assessment of their comparative performance, there is considerable uncertainty in the field about which strains to use for which applications. Different published CRISPR/Cas strategies for introducing knock-in mutations with homology-directed repair (HDR)—a key application of this technology in *Drosophila*— also have not been compared systematically.

It is also not known how many genomic target sites can be effectively edited in CRISPR/Cas experiments because most previous studies have used a small number of gRNAs. gRNAs delivered by injection of RNA or a plasmid can differ substantially in their activity (Bassett *et al.* 2013; Lee *et al.* 2014; Ren *et al.* 2014; Zhang *et al.* 2014), prompting some to recommend screening for active gRNAs in cell culture before performing whole animal experiments (Bassett and Liu 2014; Zhang *et al.* 2014).

Gantz and Bier recently described a novel CRISPR-based method, the mutagenic chain reaction (MCR), which in principle allows highly efficient somatic and germline mutagenesis of the *Drosophila* genome (Gantz and Bier 2015). The authors demonstrated using the *yellow* (*y*) gene that inserting a cassette encoding Cas9 and gRNA into the endogenous target site of a gRNA will autocatalyze its integration into the homologous allele, thus converting heterozygous into homozygous cells. Autocatalytic homing endonucleases have been created previously and are commonly referred to as “gene drives” (Chan *et al.* 2011; Windbichler *et al.* 2011; Simoni *et al.* 2014), but Gantz and Bier’s method works particularly efficiently because of the use of CRISPR/Cas. CRISPR/Cas gene drive systems could be used potentially to spread traits within wild populations of plants and animals to address global problems in public health, sustainable agriculture, and environmental management (Burt 2003; Sinkins and Gould 2006; Esvelt *et al.* 2014). Gantz and Bier (2015) proposed that their MCR technology also could be used to accelerate laboratory genome engineering and reveal homozygous mutant phenotypes in genetic screens. However, there are major biosafety concerns about the use of autonomous gene drive technology in the laboratory because of the risk of accidental infiltration of autocatalytic alleles into wild populations (Esvelt *et al.* 2014; Oye *et al.* 2014).

Here we describe the systematic evaluation of *Drosophila* CRISPR/Cas tools and experimental designs that do not create gene drives. Our findings allow evidence-based choices of Cas9-expressing lines and methods for generating knock-in alleles. Our work also reveals that optimized CRISPR with independent, integrated Cas9 and gRNA transgenes can achieve remarkably efficient germline and somatic gene targeting at a very large proportion of genomic target sites. The performance of these methods is comparable with that reported for MCR technology. We conclude that transgenic CRISPR/Cas is a safe and consistently efficient method for somatic and germline modification of the fly genome and encourage others to apply similar technology to other experimental model organisms.

## Materials and Methods

### Plasmid construction

All primer sequences [purchased from Integrated DNA Technologies (IDT)] are listed in the Supporting Information, File S1. Unless stated otherwise, enzymatic reactions were performed according to the manufacturers’ guidelines. To generate gRNA expression vectors, *pCFD3* (Port *et al.* 2014) (Addgene 49410) or *pDCC6* (Gokcezade *et al.* 2014) (Addgene 59985) were cut with *BbsI* and dephosphorylated with alkaline phosphatase, followed by gel purification of the linear plasmid. To introduce the target-specific spacer sequence, two oligonucleotides containing the spacer sequence and reverse complement spacer sequence, as well as appropriate overhangs, were mixed [1 μL of each oligo (100 μM stock solutions)] together with 1 μL of 10× T4 Ligation Buffer [New England Biolabs (NEB)], 6.5 μL of dH_2_O and 0.5 μL of T4 polynucleotide kinase (NEB). Phosphorylation and annealing of oligos was performed in a thermo cycler (30 min at 37°, 5 min at 95°, followed by ramping down to 25° at 5°/min). Annealed oligos were diluted 1:200 in dH_2_O and ligated into the linear expression plasmids with T4 DNA ligase (NEB), followed by transformation into chemically competent bacteria. A step-by-step protocol is available from www.crisprflydesign.org.

### *Drosophila* culture

Flies were maintained at 25° and 50% humidity with a 12-hr light/dark cycle.

### Assessing activity of *cas9* lines

To assess *cas9* line performance in targeting *ebony* (*e*), virgin females from the various *cas9* lines were mated to *U6:3-gRNA-e* transgenic males. The resulting double transgenic offspring were examined for CRISPR/Cas-induced somatic phenotypes, with germline mutagenesis assessed by mating randomly selected virgin females to *e* mutant males (*w;;TM3/TM6b*). The number of progeny from these crosses with ebony pigmentation was recorded. At least five independent crosses were analyzed for each *cas9* line. For these and other experiments, information on sample sizes is given in the appropriate figure legend.

To analyze the ability of the various *cas9* lines to mediate mutagenesis of *wntless* (*wls*), virgin *cas9* females were crossed to *U6:3-gRNA-wls* transgenic males. At least three independent crosses were performed for each genotype. In cases in which the resulting *cas9 U6:3-gRNA-wls* animals had significant rates of pupal lethality, the number of dead *vs.* total pupae was recorded. Males from the remaining, viable *cas9 U6:3-gRNA-wls* genotypes were crossed to *y w hs-FLP;;MKRS/TM6b* virgins. To determine the rate of germline transmission, genomic DNA was isolated from some of the offspring [using 10 μL of microLysis-Plus (Microzone) according to the manufacturer’s instructions] and used for PCR analysis. Primers *wls_geno_fwd* and *wls_geno_rev* were used to amplify the regions flanking the *gRNA-wls* target site. The genomic sequence from 10 to 12 flies from two independent crosses was analyzed for each genotype. To monitor embryonic viability of the offspring, virgins heterozygous for both *cas9* and *U6:3-gRNA-wls* were crossed to wild-type males, and the cross was transferred to cages mounted on apple juice agar plates. All embryos from a 1- to 2-hr egg collection were counted and kept at 25° for 48 hr to allow embryonic development to be completed. After 48 hr, the number of embryos that hatched was recorded. Where inspected, arrested embryos had segmentation defects. This analysis was carried out in quadruplicate for each genotype. Intercrosses of *act-cas9 U6:3-gRNA-e* flies were used as a control.

### Embryo injections and transgenesis

Microinjection into embryos was performed via standard procedures as described previously (Port *et al.* 2014). To generate transgenic gRNA lines, plasmids were often injected in pools (Bischof *et al.* 2013). Equal amounts of gRNA plasmids (5–20 per pool) were mixed and diluted to a final concentration of 150 ng/μL in dH_2_O and injected into *y v nos-PhiC31*; *attP40* [Bloomington Stock (BL)25709] embryos. A small proportion of microinjections for *Drosophila* transgenesis were performed by the fly facility injection service of the Department of Genetics, University of Cambridge, United Kingdom. Single transgenic offspring, selected by the *v^+^* marker encoded by the gRNA plasmid, were mated with *v*; *Sp/CyO* flies for several days and then squashed in 10 μL of microLysis-Plus (Microzone) to extract genomic DNA. To identify the integrated gRNA plasmid a region of the transgene was amplified by PCR using primers *U63fwd1* and *CFD4seqrev3*, and the resulting PCR product was submitted for Sanger sequencing (Source Bioscience) using the former primer.

### Assessing activity of the 66 gRNA transgenes

Transgenic gRNA males were crossed to *act-cas9* virgin females. Crosses with gRNAs targeting essential genes were monitored daily, with the proportion of dead offspring estimated at each developmental stage. The activity of gRNAs targeting nonessential genes was monitored in adult offspring of *cas9* x *gRNA* crosses (note that we did not notice significant lethality before adulthood in any of these crosses). Flies expressing *act-cas9* and gRNAs targeting *sepia* (*se*) were analyzed 1 wk after eclosion, when the difference in eye pigmentation between wild-type and *se* mutant tissue was most obvious. The percentage of eye tissue that was *se* mutant was analyzed in at least 20 *act-cas9 gRNA-se* flies by three researchers blind to the genotype. The results were very similar for each researcher and the average of the three independent recordings is presented in Figure 3. Flies expressing gRNAs targeting *y* or *e* and *act-cas9* were inspected visually for their respective pigmentation phenotype between 3 and 6 d after eclosion. Male flies were selected independently of the severity of the somatic pigmentation phenotype and crossed to either *y* or *e* mutant partners. The number of progeny with yellow or ebony pigmentation was recorded. Data presented in Figure 3 are the average of three independent crosses. The percentage of yellow offspring was normalized to account for the 50% of flies that were expected to be yellow without CRISPR mutagenesis due to the parental genotype.

### Image acquisition and processing

Flies were anesthetized with CO_2_ and submerged in 90% ethanol/10% glycerol for at least 4 hr, followed by mounting on Sylgard plates (Dow Corning) in 50% ethanol/50% glycerol. Images were captured with a Canon 550D camera equipped with a Canon 24 mm f1.8 lens mounted on a stereomicroscope (Leica MZFLIII). Camera settings were entirely manual and constant illumination was used in each session. Images presented in the same figure were captured in a single session. Brightness and contrast was adjusted with Adobe Photoshop software, with identical manipulations for each image within a series.

### Sequence analysis of CRISPR/Cas-induced mutations

Genomic DNA from single flies was extracted with 10 μL of microLysis-Plus. Then, 0.75 μL of DNA solution was used as a template in 25 μL of polymerase chain reactions (PCRs) using the Q5 Hot Start 2x master mix (NEB). Sequences of primers used for the individual target genes are listed in the File S1. PCR products were purified with the QIAGEN PCR purification kit and submitted to Sanger sequencing using the forward PCR primer. Sequencing chromatograms from flies that inherited a CRISPR/Cas-induced indel and are consequently heterozygous at the gRNA target site contained an overlay of sequence from both alleles. Chromatograms were analyzed with Tide [http://tide.nki.nl/ (Brinkman *et al.* 2014)] and re-examined manually to correct for possible mistakes by the software, which occurred occasionally for alleles involving insertions.

### Assessing different HDR strategies

Primers *pBSwg3′HA_fwd* and *pBS-wg5′HA_rev* were used to amplify the 5′ and 3′ *wingless* (*wg*) homology arms and the pBluescript SK-(+) vector backbone from the previously described *wg*::*GFP* donor plasmid (Port *et al.* 2014). The *3xP3-RFP-αtub-3′UTR* (a gift from Nick Lowe and Daniel St Johnston) sequence was amplified by PCR using primers *3xP3-RFP_fwd* and *3xP3-RFP_rev*. Both fragments were then joined by Gibson assembly to generate the red fluorescent protein (RFP) donor construct. Sequence-verified plasmid DNA was purified using the MinElute PCR purification kit from QIAGEN. To test HDR efficiency using transgenic *cas9* and gRNA, the purified circular donor plasmid was injected into embryos resulting from crosses between *nos-cas9* CFD2 or *nos-cas9* TH_attP2 virgin females and *U6:3-gRNA-wg* males. For the protocol based on transgenic *cas9* stocks, both the donor and *U6:3-gRNA-wg* plasmid were injected into embryos either hemizygous (CFD2 males) or homozygous (CFD2 females or TH_attP2 males and females) for the *nos-cas9* transgene. To test HDR efficiency using a derivative of the *pDCC6* plasmid (Gokcezade *et al.* 2014) encoding *cas9* and *U6:2-gRNA-wg* (hereafter called *pDCC6-wg*), both donor and *pDCC6-wg* were injected into embryos of an isogenized *w^1118^* stock (Bloomington Stock Centre ((BL)5905)). The concentrations of injected plasmids for the HDR experiments are given in Figure 2. Injected embryos were kept at 18° for ∼48 hr and then moved to 25°. G_0_ males were crossed to *yw hs-FLP*; *Sp/CyO* females, and the resulting male offspring was screened for the presence of RFP. To test whether the *3xP3-RFP* cassette was inserted into the *wg* locus, genomic DNA was isolated from some of the RFP positive male offspring for PCR analysis as described above, with primer pairs *wgHRgeno_fwd1/wgHRgeno_rev1* and *wgHRgeno_fwd2*/*wgHRgeno_rev2* used to amplify the regions flanking each homology arm.

## Results

### Publicly available Cas9-expressing strains vary widely in their pattern and level of activity

We and others have shown that strains with integrated *cas9* transgenes can be used to modify the *Drosophila* genome in somatic and germline cells (Sebo *et al.* 2013; Kondo and Ueda 2013; Ren *et al.* 2013, 2014; Gratz *et al.* 2014; Port *et al.* 2014; Zhang *et al.* 2014; Xue *et al.* 2014; Chen *et al.* 2015). However, there is significant uncertainty in the fly community about which of the many published *cas9* strains are most effective for genome engineering experiments.

**Figure 1:**
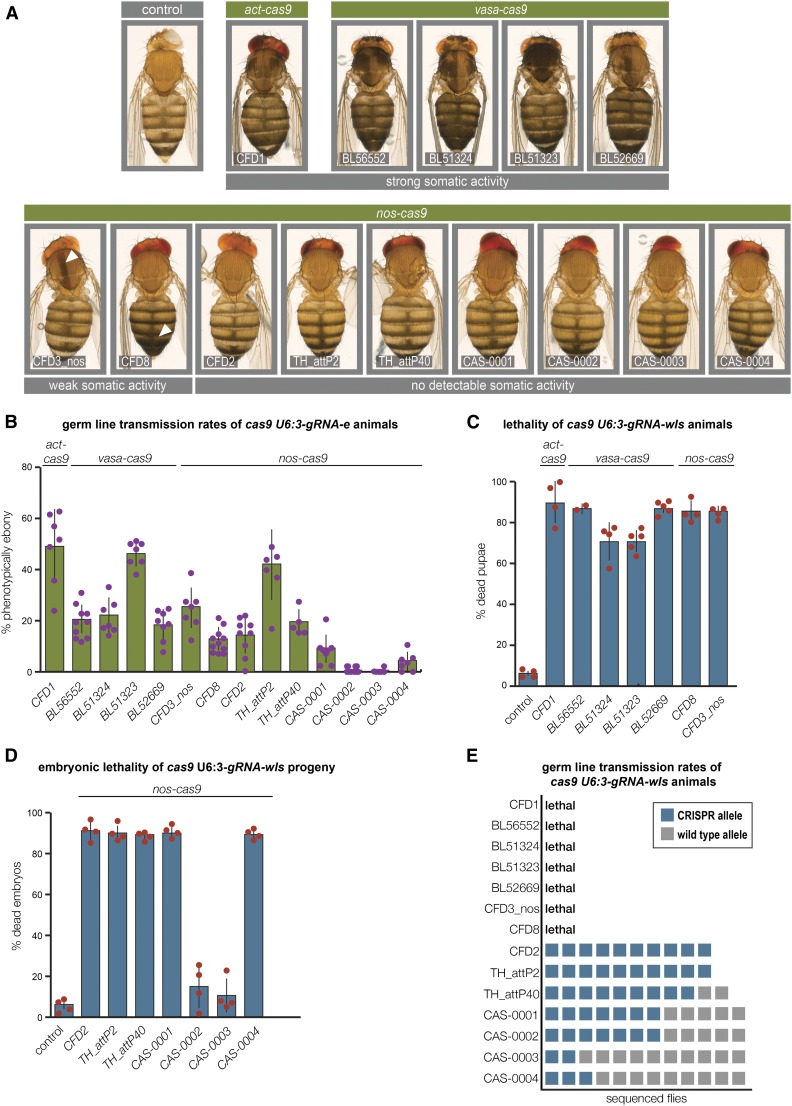
Large variation in somatic and germline activity of publicly available transgenic *cas9* strains. (A) Representative examples of female flies expressing one copy of the different *cas9* transgenes and one copy of *U6:3-gRNA-e* transgene (>100 adults of each genotype were examined). Darker body coloration indicates biallelic disruption of *e* in epidermal cells (arrowheads: sporadic biallelic targeting of *e*). (B) Assessment of germline transmission of nonfunctional *e* alleles from animals expressing different *cas9* transgenes and the *U6:3-gRNA-e* transgene. In (B–D), dots show data from individual crosses with mean ± SD represented by the underlying bar chart. In (B), typically between 30 and 140 progeny were analyzed for each cross. (C) Frequency of pupal lethality caused by *cas9* transgenes with somatic activity in combination with the *U6:3-gRNA-wls* transgene. Flies that eclosed from the pupal case had leg and wing defects consistent with reduced *wls* function and died shortly afterward. In (C) and (D), control flies were of the genotype *act-cas9 U6:3-gRNA-e*, which have widespread induction of double-strand breaks. Typically, 50–150 animals were examined for each cross. (D) Percentage of nonviable embryos laid by females expressing different germline restricted *nos-cas9* transgenes and the *U6:3-gRNA-wls* transgene following mating to wild-type males. Typically, 100 – 300 embryos were examined for each cross. (E) Germline transmission rates of CRISPR/Cas-induced *wls* mutations. Offspring from *nos-cas9 U6:3-gRNA-wls* male flies crossed to wild-type females were genotyped by polymerase chain reaction of a region of the *wls* locus containing the target site, followed by sequencing. Each square represents an individual genotyped fly.

We evaluated the performance of all transgenic *cas9* lines available at the time of study. The collection consisted of six lines that we assessed previously (Port *et al.* 2014) and eight others (Table 1). The transgenes differed in regulatory elements, *cas9* codon usage, and genomic integration sites (Table 1). We crossed *cas9* flies to a previously validated, ubiquitously expressed gRNA transgene (*U6:3-gRNA-e)*, which targets the 5′ end of the coding sequence of the pigmentation gene *ebony* (*e*) (Port *et al.* 2014). Phenotypic assays using this gRNA typically report only on out-of-frame indels, as most in-frame mutations at the *gRNA-e* target site retain protein function (Port *et al.* 2014). All adult progeny expressing *U6:3-gRNA-e* and *cas9* under the control of either the *actin5c* (*act*) or *vasa* promoter had a large proportion of cuticle that was dark, demonstrating efficient biallelic disruption of *e* in somatic cells (Figure 1A). In contrast, seven of the nine lines tested that express *cas9* under *nanos* (*nos*) regulatory elements did not have detectable somatic activity when crossed to *U6:3-gRNA-e* [Figure 1A; the other two lines (CFD3_nos and CFD8) resulted in small somatic mutant clones as previously reported (Port *et al.* 2014)]. Collectively, these findings confirm and extend our previous findings on differential somatic activity of Cas9 with these classes of regulatory elements (Port *et al.* 2014).

**Table 1:** Transgenic *cas9* lines used in this study

Construct(s)	Name (s)	Cas9 Coding Sequence*a* (Addgene Plasmid Number)	Integration Site of *cas9* Construct (Chromosome Arm)	Reference
*act-cas9*^b^	CFD1; BL54590	Hs_Cas9 (41815, 62209)	attP-ZH2a (X)	Port *et al.* (2014)
*vasa-cas9*	BL56552	Hs_Cas9 (42230)	attP-VK00037 (2L)	Gratz *et al.* (2014)
*vasa-cas9*^b^	BL51324	Hs_Cas9 (42230)	attP-VK00027 (3R)	Gratz *et al.* (2014)
*vasa-cas9*^b^	BL51323	Hs_Cas9 (42230)	attP-ZH2a (X)	Gratz *et al.* (2014)
*vasa-cas9*	BL52669	Hs_Cas9 (41815)	attP-ZH2a (X)	Sebo *et al.* (2013)
*nos-cas9*^b^	CFD2; BL54591	Hs_Cas9 (41815, 62208)	attP-ZH2a (X)	Port *et al.* (2014)
*nosG4VP16 UAS-cas9*^b^	CFD3_nos; BL54593	Dm_Cas9	attP2 (3L)	Port *et al.* (2014)
*nos-cas9:GFP*^b^	CFD8	Hs_Cas9:GFP (42234)	attP-ZH2a (X)	Port *et al.* (2014)
*nos-cas9*	TH00787.N	Dr_Cas9	attP2 (3L)	Ren *et al.* (2013)
*nos-cas9*	TH00788.N	Dr_Cas9	attP40 (2L)	Ren *et al.* (2013)
*nos-cas9*	CAS-0001	Hs_Cas9 (41815)	attP40 (2L)	Kondo and Ueda (2013)
*nos-cas9*	CAS-0002	Hs_Cas9 (41815)	Random insertion (X)	Kondo and Ueda (2013)
*nos-cas9*	CAS-0003	Hs_Cas9 (41815)	Random insertion (3)	Kondo and Ueda (2013)
*nos-cas9*	CAS-0004	Hs_Cas9 (41815)	Random insertion (2 (CyO balancer))	Kondo and Ueda (2013)

*act*, *actin5C*; *nos*, *nanos*; BL, Bloomington *Drosophila* Stock Center stock number; Hs, *Homo sapiens*; Dm, *Drosophila melanogaster*; Dr, *Danio rerio*.

a Species refers to codon optimization; all constructs express *Streptococcus pyogenes* Cas9 protein.

b Lines that were also evaluated in Port *et al.* (2014).

We next tested to what extent each *cas9* line could induce heritable mutations in the germline. *cas9 U6:3-gRNA-e* flies were crossed to *e* mutants, allowing transmission of CRISPR/Cas-induced *e* loss-of-function alleles to be evaluated by the pigmentation of the offspring. Mean germline transmission rates of nonfunctional *e* alleles varied widely for the different *cas9* lines, with values between 0.5 and 49% (Figure 1B). *act-cas9*, *vasa-cas9* BL51323, and *nos-cas9* TH00787.N (which we refer to as TH_attP2) gave rise to similar, high rates of mutagenesis.

We also evaluated the *cas9* lines with a second gRNA transgene (*U6:3-gRNA-wls*) targeting the essential gene *wntless* (*wls*) (Port *et al.* 2014). As expected, *cas9* transgenic strains that had somatic activity in combination with *U6:3-gRNA-e* resulted in nonviable progeny when crossed to *U6:3-gRNA-wls* (Figure 1C). The progeny of the other seven *nos-cas9* lines were viable in combination with *U6:3-gRNA-wls*, although a small proportion of adults from these crosses had wing defects indicative of gene targeting in a small subset of somatic cells (Figure S1). Targeting of *wls* in the germline was assessed by crossing *nos-cas9 U6:3-gRNA-wls* females to wild-type males. All but two of the *nos-cas9* transgenes resulted in a high proportion of embryos arresting with defective segmentation (Figure 1D), a phenotype associated with biallelic disruption of *wls* in the female germline (Bänziger *et al.* 2006; Bartscherer *et al.* 2006). Thus, the majority of *nos-cas9* lines can be used to assess the embryonic phenotype associated with disruption of an essential gene in the female germline.

All crosses of *nos-cas9 U6:3-gRNA-wls* females to wild-type males gave rise to some viable offspring, with sequencing of the *wls* locus from these flies revealing substantial variation in the frequency of germline transmission of CRISPR/Cas-induced mutations (Figure 1E). In two cases, CFD2 and TH_attP2, all analyzed offspring (10/10) had an indel at the *wls* target site. TH_attP40 also resulted in very efficient targeting, with 9/11 progeny inheriting a modified *wls* allele. Many inherited *wls* mutations were out-of-frame, presumably disrupting gene function. For females expressing *U6:3-gRNA-wls* and the *nos-cas9* CAS series of lines there was not a strong correlation between the rate of embryonic lethality of the progeny (Figure 1D) and the rate of germline transmission of mutated *wls* alleles (Figure 1E). Germline transmission depends on a mutation of *wls* in the transcriptionally quiescent oocyte nucleus, whereas embryonic lethality arises from mutagenesis of the gene in the auxiliary nurse cells, which supply protein products to the egg through cytoplasmic bridges. These *nos-cas9* lines may therefore be differentially active in targeting *wls* in the nurse cells and oocyte within the female germline.

Together our results reveal that the *cas9* lines differ substantially in the pattern and level of their activity. Whereas *act-cas9* and *vasa-cas9* lines can directly reveal null mutant phenotypes in the soma when combined with gRNA transgenes, a subset of *nos-cas9* lines (*e.g.*, CFD2, TH00788.N (referred to as TH_attP40) and TH_attP2) are ideally suited to efficiently generate indel mutations in both essential and nonessential genes in the germline.

### Assessment of methods for generating knock-in alleles using CRISPR/Cas

Another important application of CRISPR/Cas is the precise modification of the genome by HDR. This involves Cas9-mediated induction of double-strand breaks at the target site in the presence of exogenous donor DNA. Three experimental strategies are particularly appealing in *Drosophila* because of their relative simplicity. Embryos that are transgenic for both *cas9* and *gRNA* can be injected with donor DNA (Port *et al.* 2014), transgenic *cas9* embryos can be injected with a mixture of donor DNA and gRNA-encoding plasmid (Gratz *et al.* 2014; Ren *et al.* 2014; Zhang *et al.* 2014) or nontransgenic embryos can be injected with a donor plasmid together with a plasmid encoding Cas9 and gRNA (Gokcezade *et al.* 2014). No study has directly compared the efficiency with which all three methods facilitate HDR with the same gRNA and donor construct.

**Figure 2:**
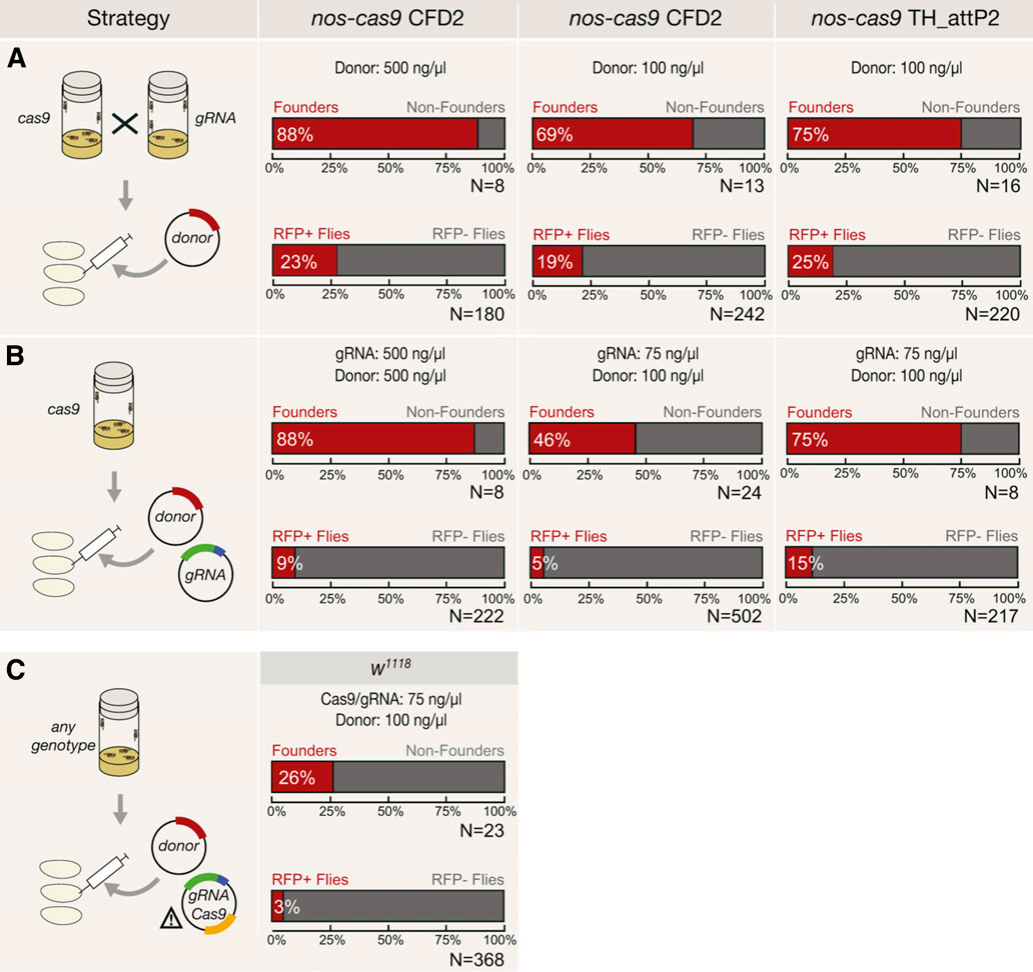
Assessing strategies for generating knock-in alleles with CRISPR/Cas-mediated HDR. (A) *nos-cas9* female flies of the indicated strain were crossed to *U6:3-gRNA-wg* males and a donor plasmid encoding RFP was injected into the embryonic progeny at the indicated concentrations. Due to a pre-existing X-linked RFP insertion in the CFD2 stock only male G_0_ and F_1_ flies could be analyzed. For consistency we only analyzed male flies in all other HDR experiments. G_0_ males that give rise to RFP-positive offspring were designated “founders.” The percentage of male progeny with RFP expression relative to all offspring (*i.e.*, from fertile founder and nonfounder G_0_ males) is indicated below. N, total number of males analyzed. (B) Donor and *U6:3-gRNA-wg* plasmids were injected into *nos-cas9* embryos. In (B) and (C), plasmids were mixed to give an injection solution containing the concentrations shown. (C) Nontransgenic *w^1118^* embryos were injected with donor DNA and a single plasmid containing both *hsp70-cas9* and *U6:2-gRNA-wg*. The attention sign indicates that unintended integration of this plasmid at the gRNA target site could create an autonomous gene drive. Propagation of a gene drive is not possible in our experiment as integration at the *gRNA-wg* target site would create a lethal allele. Table S1 contains detailed results for all HDR experiments.

To address this issue, we constructed a donor plasmid designed to knock-in a cassette expressing RFP under an eye specific promoter into the essential *wg* gene (Figure S2A). The plasmid, which contained *wg* homology arms of 1.4 and 1.7 kb, was used in circular form in combination with a previously validated gRNA [*gRNA-wg* (Port *et al.* 2014)]. The 3xP3-RFP cassette allows rapid identification of integration events by screening adults for red fluorescent eyes (Figure S2B). For technical reasons (see the legend of Figure 2) we only followed integration of donor DNA using male flies.

For the experiments involving transgenic *cas9* supply we used different plasmid concentrations and two different *nos-cas9* strains (CFD2 and TH_attP2; Figure 2, A and B). Injection of the donor plasmid into *cas9 gRNA-wg* double transgenic embryos resulted in 19–25% of all offspring from G_0_ flies having integration of the RFP construct, compared with 5–15% when the *U6:3-gRNA-wg* plasmid was injected with donor DNA into *nos-cas9* embryos (Figure 2, A and B and Table S1).

We assessed the efficiency of plasmid-based delivery of Cas9 and gRNA using a published vector that encodes both components (Gokcezade *et al.* 2014). Although expected to be a very rare event, accidental integration of such a plasmid at the gRNA target site could create an autonomous gene drive. We were able to safely test this plasmid as insertion at the *gRNA-wg* target site would be homozygous lethal, preventing the allele from being propagated. After coinjection of the dual *cas9*/*gRNA* plasmid and donor plasmid into nontransgenic embryos only 3% of all progeny from G_0_ flies had genomic integration of the RFP cassette (Figure 2C and Table S1). Integration of the donor DNA within the *wg* locus could be confirmed for 87 of 98 RFP-positive flies tested from our entire set of HDR experiments (Figure S2, C and D and Table S1). In summary, the methods in which a transgenic *cas9* source was used resulted in the greatest frequency of knock-in alleles, with injection into embryos that are also transgenic for the gRNA giving the most efficient targeting.

### CRISPR-it with a highly active Cas9 line results in remarkably efficient somatic and germline mutagenesis at a large proportion of target sites

The aforementioned results, together with previous literature (Kondo and Ueda 2013; Port *et al.* 2014), provide evidence that transgenic supply of both Cas9 and gRNA can lead to very high rates of mutagenesis. An important outstanding question is whether the high rates of efficiency observed for a small number of genomic target sites in these studies are a general feature of this system.

To determine what fraction of gRNAs is functional in the CRISPR-it system we generated a set of 66 transgenic fly strains each expressing a different gRNA. The gRNAs were designed to target seven genes, including essential genes [*wingless* (*wg*), *wls*, *Lissencephaly-1* (*Lis1*), *Dynein heavy chain at 64C* (*Dhc64C*)] and nonessential pigmentation genes [*e*, *y*; also controlling coloration of the cuticle, and *sepia* (*se*; controlling coloration of the eye)]. gRNA target sites were based on the reference sequence of the *Drosophila* genome (Flybase release 6.02). They were located in the 5′ half of the coding sequence of each gene, such that out-of-frame indels are likely to create loss-of-function alleles, but were otherwise selected at random. gRNAs were expressed under the control of the strong, ubiquitous *U6:3* promoter (Port *et al*., 2014), with all constructs integrated at the same genomic location (attP40 on chromosome 2L) to ensure comparable expression.

**Figure 3:**
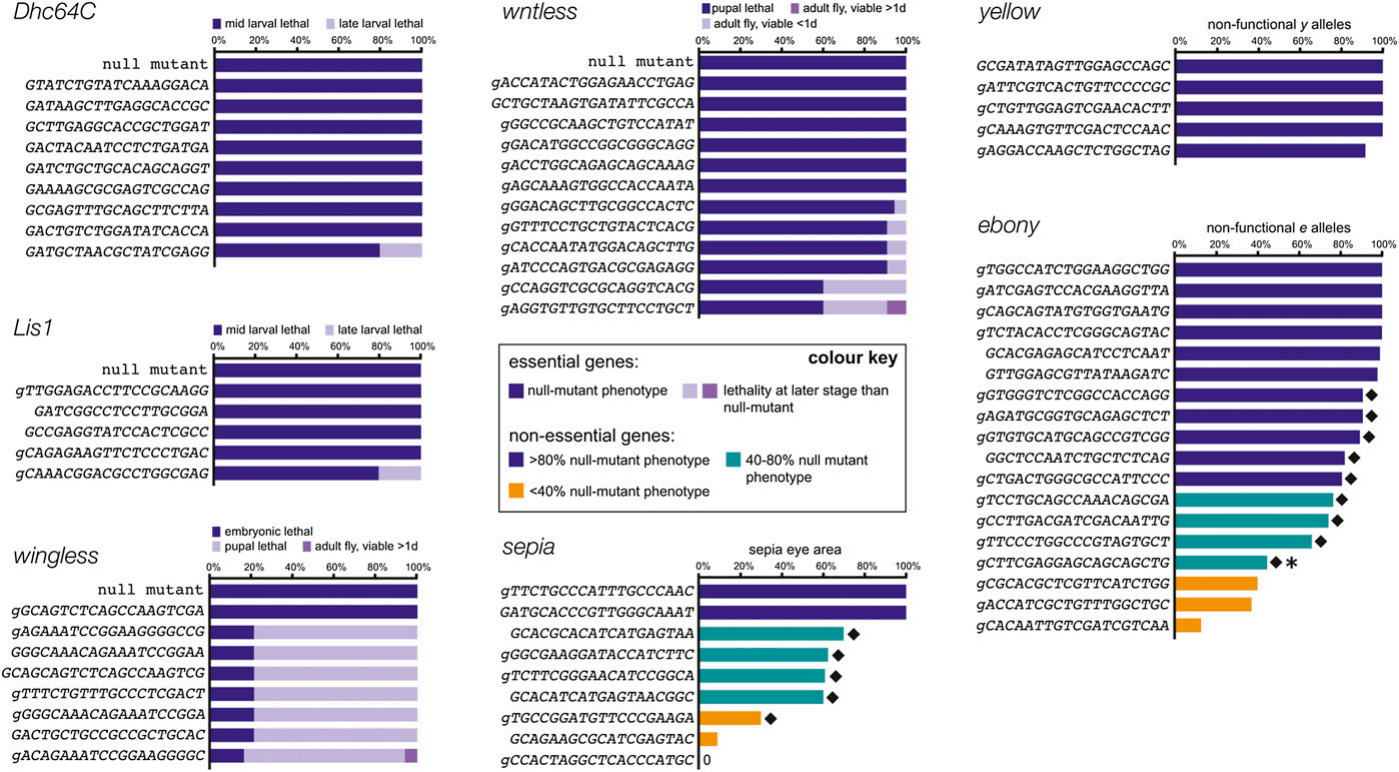
Almost all transgenic gRNAs are highly active with a strong transgenic source of Cas9. Male flies transgenic for individual gRNAs were crossed to *act-cas9* females. Somatic phenotypes of *act-cas9 gRNA* progeny are summarized for gRNAs targeting *Dhc64C*, *Lis1*, *wg*, *wls*, and *se*. The *gRNA-se* with no detectable activity was found to contain a polymorphism. Activity in the germline is shown for gRNAs targeting *y* and *e* [data show mean percentage of progeny that inherited a nonfunctional allele from three independent crosses (see Table S2 for more details and somatic phenotypes induced by these gRNAs)]. gRNAs highlighted by black diamonds frequently give rise to functional in-frame mutations (Table S2), suggesting that their efficiency in creating indel mutations often approaches 100%. The gRNA additionally highlighted by an asterisk gives rise to an unexpected large number of in-frame mutations in functional alleles analyzed (8/8 flies), suggesting a micro-homology-mediated bias in nonhomologous end joining. Since transcription initiation from *U6* promoters is proposed to require a G nucleotide, we extended gRNAs by a mismatched G where necessary (lower case). gRNAs with spacer sequences of 18 nt, 19 nt, and 20 nt can support efficient mutagenesis, consistent with previous evidence that short truncations of spacers usually do not strongly affect activity of gRNAs (Fu *et al.* 2014; Ren *et al.* 2014).

The 66 individual gRNA strains were crossed to the X-linked *act-cas9* line, which has ubiquitous, high activity (Figure 1, A and B). Strikingly, analysis of the progeny revealed that all but one of the gRNAs gave rise to a somatic mutant phenotype (Figure 3 and Table S2). Sequencing data from our strains revealed a single-nucleotide polymorphism in the target site of the inactive gRNA, designed to target *se*, providing a likely explanation for its inactivity. Most of the gRNAs targeting the essential genes caused lethality at the same developmental stage as reported for the respective homozygous null-mutant animals, with the remainder leading to developmental arrest at later stages (Figure 3). With the exception of the single inactive *gRNA-se* transgene, all of the gRNAs targeting nonessential genes gave adults with regions of tissue with the null-mutant pigmentation phenotype (Figure 3, Figure S3, and Table S2). For almost all of these gRNAs, biallelic gene disruption was usually observed in more than 50% of the affected tissue, with half of the gRNAs, including all five targeting *y*, leading to more than 80% mutant tissue (Table S2). Nonspecific phenotypes were not observed in *act-cas9* adults coexpressing any of the gRNA transgenes, consistent with previous evidence that CRISPR/Cas operates with substantial fidelity in *Drosophila* (Bassett *et al.* 2013; Gratz *et al.* 2014; Ren *et al.* 2014). The aforementioned results demonstrate that the vast majority of randomly selected gRNAs efficiently induce biallelic loss-of-function mutations in target genes, thereby directly revealing the somatic mutant phenotype. There was, however, variation in the strength of the phenotypes observed for gRNAs targeting the same gene (Figure 3 and Table S2). Weaker phenotypes could arise either through a reduced frequency of induced indel mutations or because functional in-frame mutations at the specific gRNA target site provide a significant fraction of cells with functional protein.

To directly test the hypothesis that in-frame mutations can mask high rates of indel induction by some gRNAs, we characterized CRISPR/Cas-induced mutations transmitted to the next generation. Flies expressing *act-cas9* and a transgenic gRNA targeting *y* or *e* were crossed to *y* or *e* mutant flies, respectively, followed by phenotypic analysis of the offspring. The rates of transmission of nonfunctional alleles across target sites was generally greater than reported when gRNAs were delivered by plasmid injection into transgenic *cas9* embryos (Ren *et al.* 2014) (Figure 3 and Figure S4, A and B), providing further evidence of the relatively high activity of CRISPR-it. All five *gRNA-y* transgenes and six of 18 *gRNA-e* transgenes transmitted nonfunctional alleles to >93% of their offspring (Figure 3). The high frequency of nonfunctional alleles strongly suggests that in-frame mutations at these target sites usually disrupt protein function. Transmission rates for nonfunctional mutations ranged from 13 to 90% for the other 12 *gRNA-e* lines (Figure 3). To explore the basis of this variation, we determined the sequence of *e* around the target sites in some of the phenotypically wild-type progeny from the 12 lines. For nine of the gRNAs, in-frame indel mutations were detected in all, or almost all, analyzed animals (Table S2 and Figure 3). Thus these gRNAs appear to create indels in the vast majority of *e* alleles, with differences in the functional effects of in-frame mutations at their target sites influencing the outcome of phenotypic assays. We confirmed that this effect was not specific for target sites in *e* by molecular analysis of the *se* locus in offspring of *act-cas9 gRNA-se* flies, which again revealed in-frame mutations in the majority of functional alleles (Table S2 and Figure 3).

Indel mutations were not found readily in functional alleles inherited from flies expressing *act-cas9* and the three *gRNA-e* transgenes that were the least efficient in the phenotypic assays (transmission of nonfunctional alleles in 13–35% of cases) or one poorly active *se* gRNA (7% of phenotypically mutant eye tissue) (Table S2 and Figure 3). Thus, these gRNAs induce indels with relatively low frequency. Sequencing of the target site for these gRNAs excluded the possibility that polymorphisms were responsible for their reduced activity. gRNAs with lower activity were not readily predicted by available online gRNA design tools and their target sites did not have a low GC content in the region proximal to the PAM (Figure S4, C–J and Table S3), a parameter that strongly predicted less active gRNAs encoded by plasmids injected into transgenic *cas9* embryos (Ren *et al.* 2014). Several gRNAs targeting sites with relatively low PAM proximal GC content had very high activity in our experiments (Figure S4, A, F, and J). Collectively, these results demonstrate that optimized CRISPR-it is a very robust genome engineering system, leading to highly effective mutagenesis at the vast majority of target sites.

## Discussion

We set out to provide a systematic evaluation of tools and design parameters for transgenic CRISPR/Cas genome engineering in *Drosophila melanogaster*. We show that selection of the *cas9* strain is critical for the successful application of this methodology, as publicly available lines vary widely in their activity in somatic and germline cells. *act-cas*9 and all available *vasa-cas9* lines can be used with transgenic gRNAs to reveal null mutant phenotypes in somatic tissues. However, somatic expression of Cas9 in these lines means that they are not suitable for germline transmission of mutations in essential genes using gRNA transgenes. *nos-cas9* TH_attP2, CFD2, and TH_attP40 are highly effective for germline mutagenesis of both the nonessential gene *e* and the essential gene *wls*, suggesting that they are the most versatile transgenic Cas9 sources for creating heritable indel mutations with transgenic gRNAs. These three *nos-cas9* transgenes are inserted on different chromosomes (Table 1) and the choice between them will depend on the specific crossing scheme of the user. CFD2 is often used in our laboratory because its location on the X chromosome means that it can easily be selected against in subsequent generations. Our work defines for the first time *nos-cas9* transgenic lines that have relatively low activity. Although these lines are less suitable for most genome engineering experiments, they may be useful when widespread biallelic targeting in the germline is problematic, such as when the target gene is essential for cell survival.

We find that success rates vary substantially between different CRISPR/Cas-based protocols for generating knock-in alleles. High efficiency is particularly important for HDR without selectable markers (which introduce unwanted sequences into the target locus and often require additional cloning steps) as in most cases integration must be detected by PCR-based analysis of genomic DNA. The rates of HDR that we and others (Yu *et al.* 2014; Gratz *et al.* 2014; Ren *et al.* 2014; Zhang *et al.* 2014) have reported when injecting donor DNA and gRNA plasmids into *cas9* transgenic embryos are sufficient to make PCR-based screens practical. Because this method is also relatively quick and can be easily be performed by commercial injection services, it is likely to be the method of choice for most applications. However, the greater efficiency achieved by injection of donor DNA into embryos transgenic for both *cas9* and the gRNA will be attractive to users who want to maximize their chances of success and minimize the amount of work that has to be spent screening for the desired insertion. It should be noted that, once generated, the transgenic gRNA strain can also be used in other Cas9-based applications, including phenotypic analysis in somatic cells either in a stochastic or tissue-specific manner (Port *et al.* 2014; Xue *et al.* 2014).

We show that use of transgenic gRNAs with a highly active Cas9-line allows highly efficient mutagenesis across a large proportion of target sites, with our analysis of germline transmission of mutations in pigmentation genes suggesting that the vast majority of gRNAs cause close to maximal rates of indel induction. Incomplete penetrance of phenotypes induced by some transgenic gRNAs is usually due to functional in-frame mutations. This problem can be circumvented in cases in which previous knowledge highlights codons that are likely to be crucial for protein function. A more general approach is to coexpress more than one gRNA using a dual gRNA vector (*e.g.*, Port *et al.* 2014). This will increases the probability of creating a nonfunctional mutant allele, in some cases by creating deletions between the gRNA target sites (Gratz *et al.* 2013; Kondo and Ueda 2013).

Importantly, our study highlights that CRISPR-it is a safe, efficient, and robust alternative to the recently reported MCR, which uses an autonomous gene drive for functional studies (Gantz and Bier 2015) (Table 2). MCR has so far only been demonstrated at a single target site in the *Drosophila y* gene, a locus often used in pioneer studies and which appears to have an unusually high susceptibility for gene targeting (Rong and Golic 2000; Gratz *et al.* 2013). MCR can rapidly reveal recessive somatic phenotypes as heterozygous mutations are efficiently converted to the homozygous state. We show here using many target sites, including five in *y*, that CRISPR-it can induce biallelic gene disruption in the soma with similar efficiency to that reported for Gantz and Bier’s MCR experiment (Figure 3 and Table S2). Because CRISPR-it uses separate integrated *cas9* and *gRNA* transgenes, mutagenesis is inactivated by breeding. This is not the case for the MCR system due to linkage of the *cas9* and *gRNA* sequences at the target locus. This means that escape of flies from the laboratory could conceivably result in rapid spread of an MCR allele in the wild, with unpredictable ecological consequences. In fact, a recent study that theoretically modeled the spread of MCR alleles under different parameters (Unckless *et al.* 2015) concluded that “there are conditions in which accidental introductions of a single individual can lead to fixation of the MCR allele even with significant fitness consequences to the individual.” A risk of creating autocatalytic alleles is in principle also associated with other plasmids encoding both Cas9 and gRNA (*e.g.*, Gokcezade *et al.* 2014), although the probability of insertion at the gRNA target site is much lower due to the absence of homology arms.

**Table 2:** Comparison of MCR and CRISPR-it technology

	Mutagenic chain reaction	CRISPR-it
Work environment	Strict physical containment:	Regular fly room and working procedures* (*suitable for work with standard transgenic organisms)
	Triple contained flies	
	Locked facilities at all times	
	Accounting for individual flies	
	Single experimenter	
CRISPR components	On one construct	Separate, integrated transgenes
Multiplexing*a*	No	Yes
Directly reveals recessive phenotypes	Yes (so far shown for a single target site)	Yes (shown for many target sites)
Efficiency of germline transmission of mutations	Up to 100%	Up to 100%
CRISPR inactivated by crossing	No	Yes
Risk of infiltrating wild and laboratory populations	Yes	No
Genotype at target site	Mosaic in all generations (mostly homozygous for MCR allele)	F1: Mosaic (mostly biallelic for indels)
		F2: heterozygous
		F3: homozygous
Time from cloning to recessive phenotype	∼25 d	∼36 d*b*
Targeting essential genes	No*c*	Yes
Tissue-specific mutagenesis	No	Yes

MCR, mutagenic chain reaction; CRISPR-it, Clustered Regularly Interspaced Short Palindromic Repeat with independent transgenes.

a Generation of multiple gRNA plasmids can be multiplexed during cloning and transgenesis. Multiplexing is not possible during MCR.

b One additional generation.

c Targeting of essential genes might be possible in the future by introducing an MCR resistant rescue construct into the MCR cassette (Gantz and Bier 2015).

Working with flies containing an MCR cassette has to be performed using very strict biosafety procedures (Table 2) (Gantz and Bier 2015). In contrast, work with independent *cas9* and gRNA transgenes can be performed in a regular fly room suitable for work with standard transgenic animals; this means that experiments can be performed much more conveniently and rapidly. Furthermore, CRISPR-it can readily reveal mutant phenotypes of essential genes and can be employed in a tissue specific manner (Port *et al.* 2014; Xue *et al.* 2014), applications that are not easy to achieve with MCR. The ability of CRISPR-it to efficiently reveal recessive mutant phenotypes associated with a large proportion of genomic target sites also makes it a highly attractive system for large scale F_1_ mutagenesis screens.

Gene drive technology has great promise for applications such as ecosystem management and pest control and may well be adopted for basic research applications (Burt 2003; Sinkins and Gould 2006; Esvelt *et al.* 2014). However, responsible use of this technology will require development of robust molecular containment strategies (Esvelt *et al.* 2014). We therefore urge researchers not to apply gene drives without these safeguards in place. Our experiments illustrate safe and effective genome engineering strategies that are already available for basic research and can be applied to any genetically tractable organism.
